# Apparent diffusion coefficient cannot predict molecular subtype and lymph node metastases in invasive breast cancer: a multicenter analysis

**DOI:** 10.1186/s12885-019-6298-5

**Published:** 2019-11-05

**Authors:** Alexey Surov, Yun-Woo Chang, Lihua Li, Laura Martincich, Savannah C. Partridge, Jin You Kim, Andreas Wienke

**Affiliations:** 1 Department of Diagnostic and Interventional Radiology, University of Ulm, Albert-Einstein-Allee 23, 89081 Ulm, Germany; 2 Department of Radiology, Soonchunhyang University Hospital, 59 Daesakwan-ro, Yongsan-gu, Seoul, 140-743 Republic of Korea; 3 Institute of Biomedical Engineering and Instrumentation, Hangzhou Dianzi University, Hangzhou, China; 4 Unit of Radiology, Institute for Cancer Research and Treatment (IRCC), Strada Provinciale 142, 10060 Candiolo, Turin, Italy; 5 Department of Radiology, University of Washington, Seattle, Washington 825 Eastlake Ave. E, G2-600, Seattle, WA 98109 USA; 6 Department of Radiology, Pusan National University Hospital, Pusan National University School of Medicine and Medical Research Institute 1-10, Ami-Dong, Seo-gu, Busan, 602-739 South Korea; 7 Institute of Medical Epidemiology, Biostatistics, and Informatics, Martin-Luther-University Halle-Wittenberg, Magdeburger Str, 06097 Halle, Germany

**Keywords:** Breast cancer, ADC, DWI, Molecular subtype (luminal a, Luminal B, HER 2, Triple negative), Ki 67

## Abstract

**Background:**

Radiological imaging plays a central role in the diagnosis of breast cancer (BC). Some studies suggest MRI techniques like diffusion weighted imaging (DWI) may provide further prognostic value by discriminating between tumors with different biologic characteristics including receptor status and molecular subtype. However, there is much contradictory reported data regarding such associations in the literature. The purpose of the present study was to provide evident data regarding relationships between quantitative apparent diffusion coefficient (ADC) values on DWI and pathologic prognostic factors in BC.

**Methods:**

Data from 5 centers (661 female patients, mean age, 51.4 ± 10.5 years) were acquired. Invasive ductal carcinoma (IDC) was diagnosed in 625 patients (94.6%) and invasive lobular carcinoma in 36 cases (5.4%). Luminal A carcinomas were diagnosed in 177 patients (28.0%), luminal B carcinomas in 279 patients (44.1%), HER 2+ carcinomas in 66 cases (10.4%), and triple negative carcinomas in 111 patients (17.5%). The identified lesions were staged as T1 in 51.3%, T2 in 43.0%, T3 in 4.2%, and as T4 in 1.5% of the cases. N0 was found in 61.3%, N1 in 33.1%, N2 in 2.9%, and N3 in 2.7%. ADC values between different groups were compared using the Mann–Whitney U test and by the Kruskal-Wallis H test. The association between ADC and Ki 67 values was calculated by Spearman’s rank correlation coefficient.

**Results:**

ADC values of different tumor subtypes overlapped significantly. Luminal B carcinomas had statistically significant lower ADC values compared with luminal A (*p* = 0.003) and HER 2+ (*p* = 0.007) lesions. No significant differences of ADC values were observed between luminal A, HER 2+ and triple negative tumors. There were no statistically significant differences of ADC values between different T or N stages of the tumors. Weak statistically significant correlation between ADC and Ki 67 was observed in luminal B carcinoma (*r* = − 0.130, *p* = 0.03). In luminal A, HER 2+ and triple negative tumors there were no significant correlations between ADC and Ki 67.

**Conclusion:**

ADC was not able to discriminate molecular subtypes of BC, and cannot be used as a surrogate marker for disease stage or proliferation activity.

## Background

Breast cancer is a major global health problem and major cause of mortality [1]. In brief, from 2006 to 2010, in non-Hispanic white women, the average annual female breast cancer incidence rate was 127.3 cases per 100,000 females [2]. Approximately 232,340 new cases of invasive breast cancer and 39,620 deaths are expected among US women each year [2]. Furthermore, breast cancer tends to be diagnosed at a younger age than other common cancers, with a median age at diagnosis of 61 years [2]. About 19% of breast cancers are diagnosed in women ages 30 to 49 years, and 44% occur among women who are age 65 years or older [3].

Radiological imaging plays a central role in the diagnosis of BC. Furthermore, different imaging modalities, especially magnetic resonance imaging (MRI), can also provide information about histopathology in BC. For example, it has been shown that rim enhancement on dynamic MRI was associated with high expression of proliferation index Ki 67 [4]. Moreover, some reports suggest that diffusion weighted imaging (DWI) may discriminate tumors with varying receptor statuses, with differences in quantitative apparent diffusion coefficient (ADC) values observed between BC subtypes [5–7]. Roknsharifi et al. found that tumors with PR negativity and oncotype score ≥ 18 (intermediate to high risk for recurrence) demonstrated significantly lower ADC values [5]. Kato et al. reported that the minimum ADC value of Luminal A carcinomas was significantly higher than those of Luminal B tumors (0.834 vs. 0.748 × 10^− 3^ mm^2^/s; *p* < 0.025) [6]. Finally, in the study of Sharma et al., triple negative tumors showed a significantly higher ADC compared to non-triple negative cancers [7].

However, according to other authors, ADC cannot discriminate BC subtypes [8].

Overall, the role of ADC in prediction of several clinically relevant histopathological features in BC needs to yet be proven because of underlying problems in the current literature. Firstly, the reported data were based only on small number of investigated tumors/patients. Secondly, most of the published studies are retrospective with suitable bias. Thirdly, as mentioned above, the reported data are very contradictory. While some authors found significant correlations between ADC and histopathology in BC, others did not.

The aim of the present study was to analyze associations between ADC and hormone receptor status in BC in a large multicenter sample.

## Methods

### Data acquisition

This study comprises data from five centers (Table 1) as follows: Department of Radiology, Soonchunhyang University Hospital, Republic of Korea (center 1) [9]; Institute of Biomedical Engineering and Instrumentation, Hangzhou Dianzi University, Hangzhou, China (center 2) [10]; Unit of Radiology, Institute for Cancer Research and Treatment, Turin, Italy (center 3) [11]; Department of Radiology, University of Washington, Seattle, Washington, USA (center 4) [12]; and Department of Radiology, Pusan National University Hospital, Pusan National University School of Medicine and Medical Research Institute, Korea (center 5) [13].

**Table 1:** MRI techniques used in the centers

Centers	MR scanners	DWI sequences and b values	ADC measure
1	1.5 T scanner (Sonata, Siemens, Erlangen, Germany)	Single-Shot Echo Planar sequence: -TR/TE: 5000/110 ms, -FOV: 320 mm, -matrix: 128 × 128, -slice thickness: 3.5-mm, − 0.7-mm slice gap, -b values: 0–1000 s/mm^2^	-Manual placed multiple ROIs; -measure by one radiologist with 10 years of experience in breast imaging; -cystic or necrotic portions of the tumour were avoided.
2	3.0 T scanner (Magnetom Verio, Siemens, Germany)	Single-Shot Echo Planar sequence: -TR/TE: 7000/85 ms, -FOV: 104 × 320 mm, -matrix 220 × 72, -slice thickness: 6 mm; b values: 50–1000 s/mm^2^	-Manual placed multiple ROIs (whole tumor measure); -measure by one radiologist; -cystic or necrotic portions of the tumour were avoided.
3	1.5 T scanner (GE Healthcare, Milwaukee, WI, USA)	single-shot echoplanar image: -TR/TE: 7000/85 ms; -FOV 340 × 340 mm, -matrix: 128× 128, slice thickness: 4 mm, b values: 0–900 s/mm^2^	-Manual placed ROI (single ROI measure); -measure by one radiologist with 13 years of experience in breast imaging; -cystic or necrotic portions of the tumour were avoided.
4	3 T scanner (Achieva Tx, Philips Healthcare, Best, Netherlands)	single-shot echoplanar image with fat suppression (spectral attenuated inversion recovery): -TR/TE: 53367000/ 61 ms, -matrix: 240 × 240, -FOV: 360 × 360 mm, -slice thickness: 5 mm, -b values: 0–800 s/mm^2^	-Manual placed ROI (single ROI measure); -measure by one radiologist with 5 years of experience in breast MR imaging; -cystic or necrotic portions of the tumour were avoided.
5	3 T scanner (Trio Tim, Siemens, Erlangen, Germany)	Single-Shot Echo Planar sequence: -TR/TE: 6600/91 ms, - matrix: 192 × 134, -FOV: 320x320mm, -slice thickness: 2 mm, b values: 0–1000 s/mm^2^	-Manual placed ROI (single ROI measure), -ADC measure by two radiologists with 5 and 2 years of experience in breast MRI in consensus, -cystic or necrotic portions of the tumour were avoided.

Inclusion criteria were as follows: histopathological diagnosis, available ADC values, hormone receptor status, Ki 67 index, and TNM stage.

### Patients, tumors and MRI

Overall, 661 patients (all female; mean age, 51.4 ± 10.5 years; median age, 50.5 years; range, 24–85 years) were included in the study. The histological type of BC was defined according to the WHO classification [14]. Invasive ductal carcinoma was diagnosed in 625 patients (94.6%) and invasive lobular carcinoma in 36 cases (5.4%). There were tumors with different hormone receptor status. The receptor status of the acquired breast carcinomas were classified according to the San Gallen Consensus Meeting [15]. Luminal A carcinomas (i.e. hormone receptor positive carcinomas with a Ki 67 expression < 14%) were diagnosed in 177 patients (28.0%), luminal B carcinomas (i.e. hormone receptor positive tumors with a Ki 67 expression > 14%) in 279 patients (44.1%), HER 2+ carcinomas in 66 cases (10.4%), and triple negative carcinomas in 111 patients (17.5%).

Well differentiated (grade 1) BC were diagnosed in 9.9%, moderately differentiated (grade 2) in 57.9% and poorly differentiated (grade 3) tumors in 32.2% of the patients. Furthermore, the identified lesions were staged as T1 in 51.3%, T2 in 43.0%, T3 in 4.2%, and as T4 in 1.5% of the cases. Regarding N stage, N0 was found in 61.3%, N1 in 33.1%, N2 in 2.9%, and N3 in 2.7%. There were no tumors with distant metastases (M stage).

In all cases, MRI with DWI was performed on 1.5 or 3.0 T clinical scanners with dedicated breast radiofrequency coils (Table 1).

### Statistical analysis

For statistical analysis the SPSS statistical software package was used (SPSS 17.0, SPSS Inc., Chicago IL, USA). Continuous variables were described by mean value, median and standard deviation. Categorical variables were given as relative frequencies. ADC values between different groups were compared using the Mann–Whitney U test (two-group comparisons) and by the Kruskal-Wallis H test (multiple-group comparisons), where the *p*-values are adjusted for multiple testing (Bonferroni correction). The association between ADC and Ki 67 values was calculated by Spearman’s rank correlation coefficient.

## Results

### ADC and molecular subtypes

**Fig. 1:**
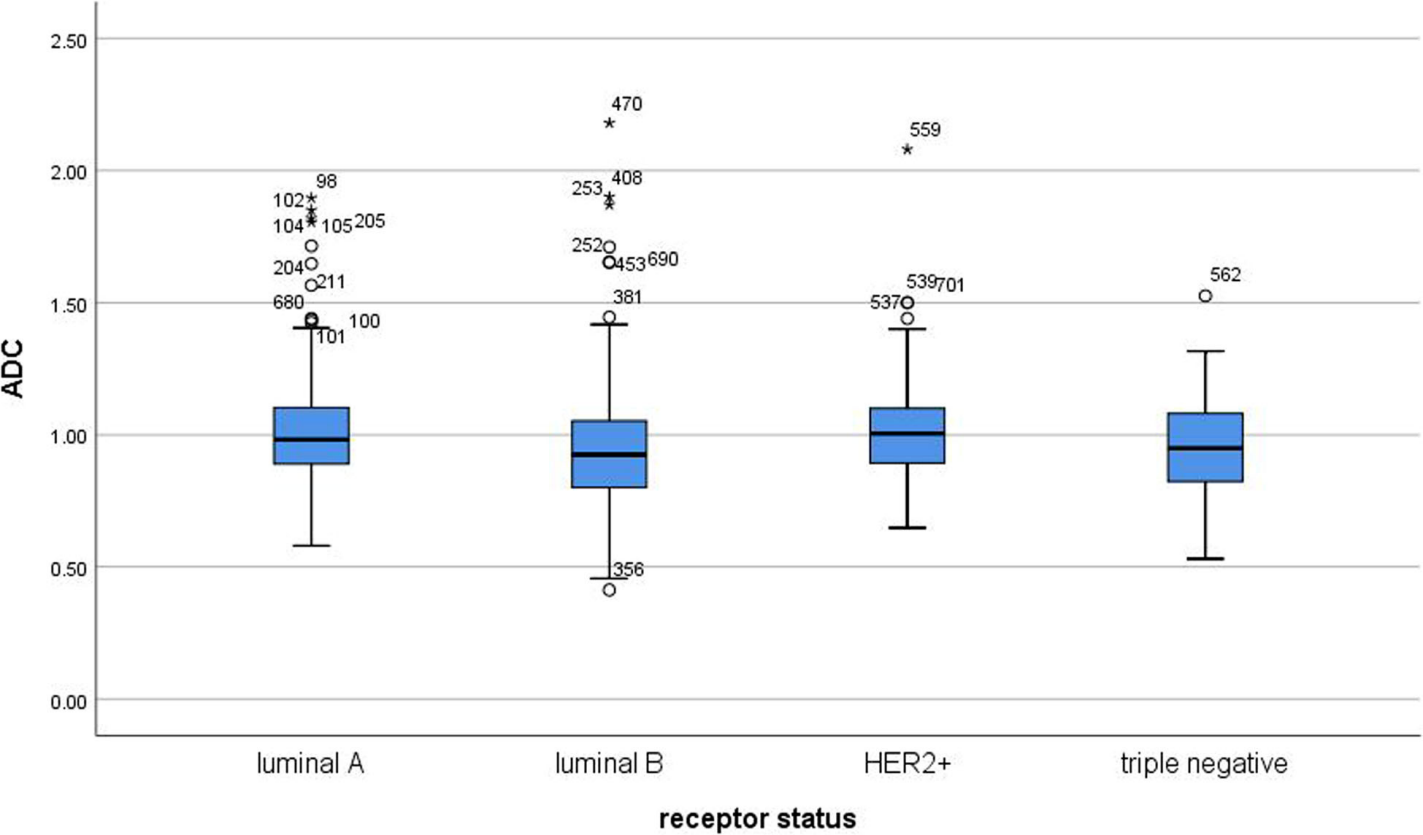
Box plots of ADC values in tumors of different molecular subtype

ADC values differed between tumors of different molecular subtype (Table 2). Luminal B carcinomas had statistically significant lower ADC values compared with luminal A (*p* = 0.003) and HER 2+ (*p* = 0.007) lesions. However, ADC values of different tumor subtypes overlapped significantly, and no significant differences of ADC values were observed between luminal A, HER 2+ and triple negative tumors (Fig. 1).

**Table 2:** Comparison of tumor ADC values between molecular subtypes

Tumors					Kruskal-Wallis test
	Luminal A, *n* = 177	Luminal B, *n* = 279	HER 2+, *n* = 66	Triple negative, *n* = 111	
ADC values, ×10^−3^ mm^2^ s^−1^, M ± SD	1.01 ± 0.22 *p* = 1.00 vs HER 2+	**0.95 ± 0.23** *p* = 0.003 vs luminal A; *p* = 0.007 vs HER 2+	1.04 ± 0.23 *p* = 0.168 vs triple negative	0.95 ± 0.17 *p* = 0.309 vs luminal A; p = 1.00 vs luminal B	*P* < 0.001

### ADC and tumor stage

**Fig. 2:**
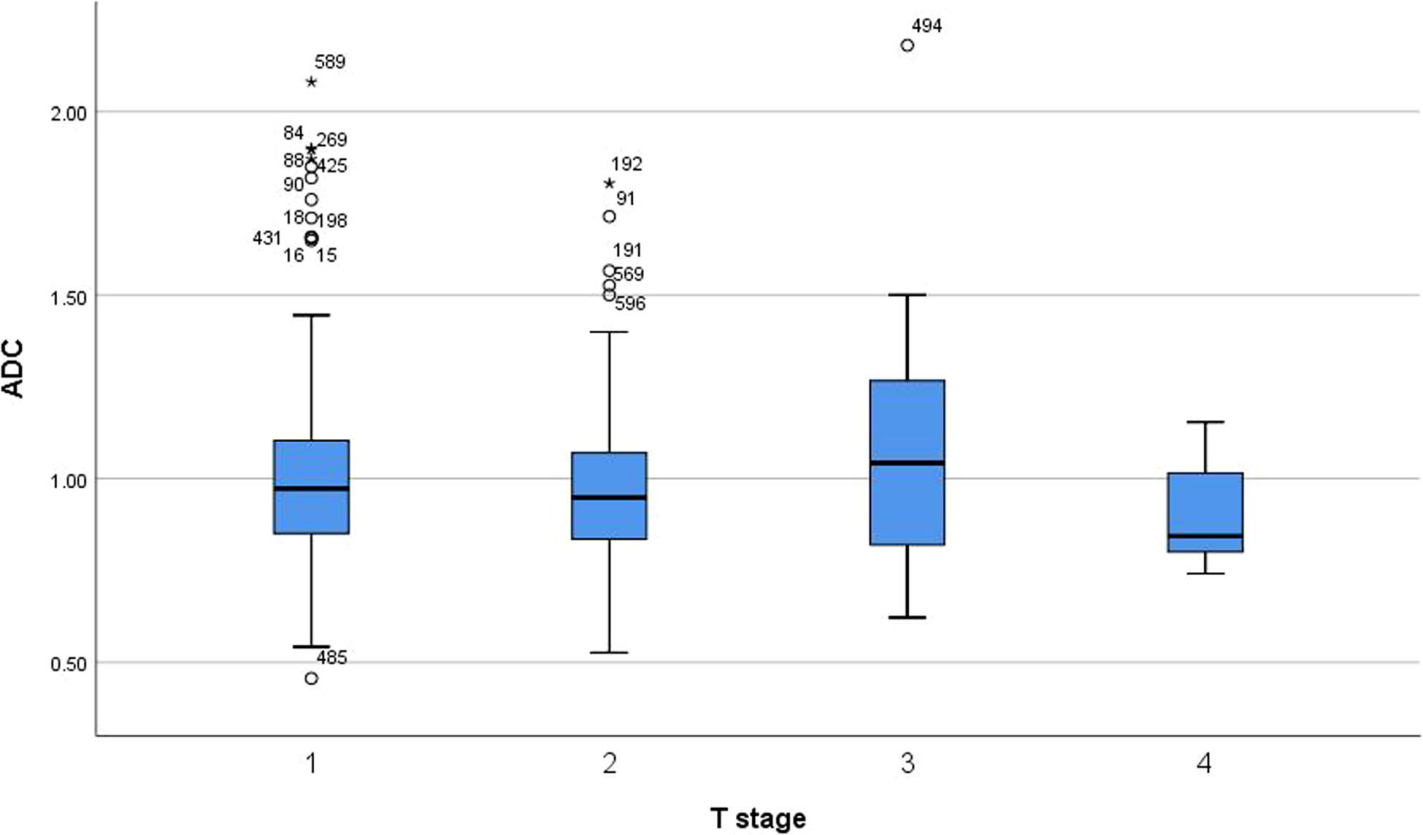
Box plots of ADC values in different stages of primary tumors. There was no statistical difference between the ADC values (Kruskal-Wallis test *p* = 0.086)

**Fig. 3:**
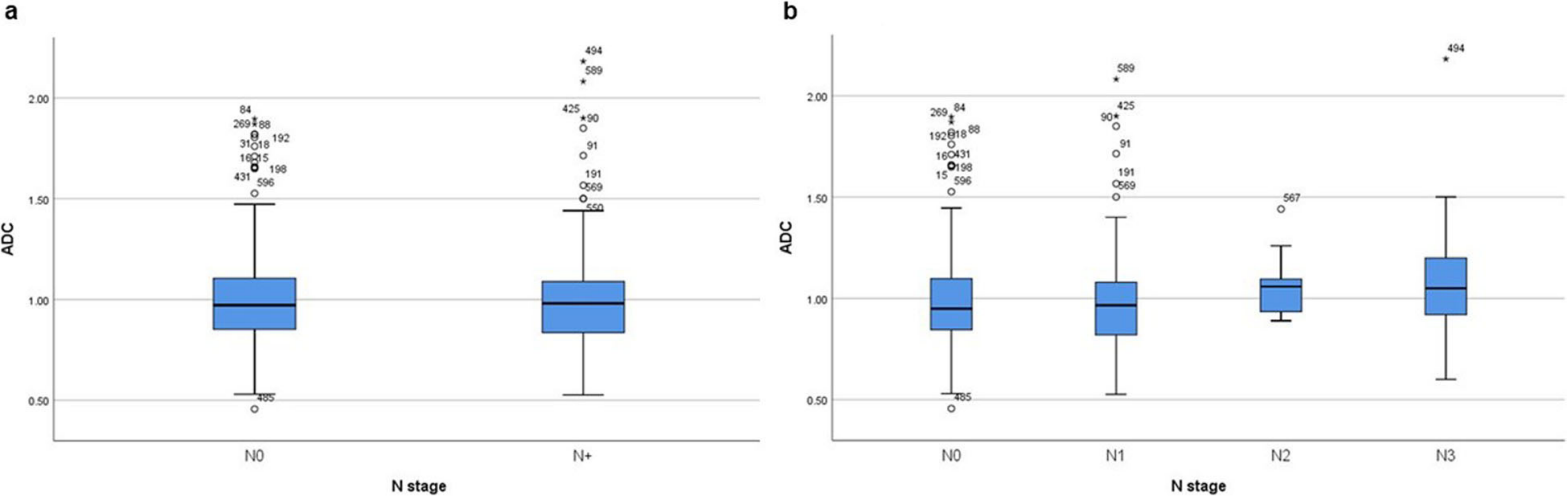
**a** Comparison of ADC values between N0 and N+ tumors. No statistical difference between the ADC values was found (*p* = 0.849). **b** Box plots of ADC values in different N stages of breast cancer. There was no statistical difference between the ADC values (Kruskal-Wallis test *p* = 0.135)

There were no statistically significant differences of ADC values between different stages of primary tumors (Fig. 2). Also ADC cannot discriminate N0 from N+ tumors (Fig. 3a). In addition, there was no difference of ADC values between N0, N1, N2 and N3 tumors (Fig. 3b).

**Fig. 4:**
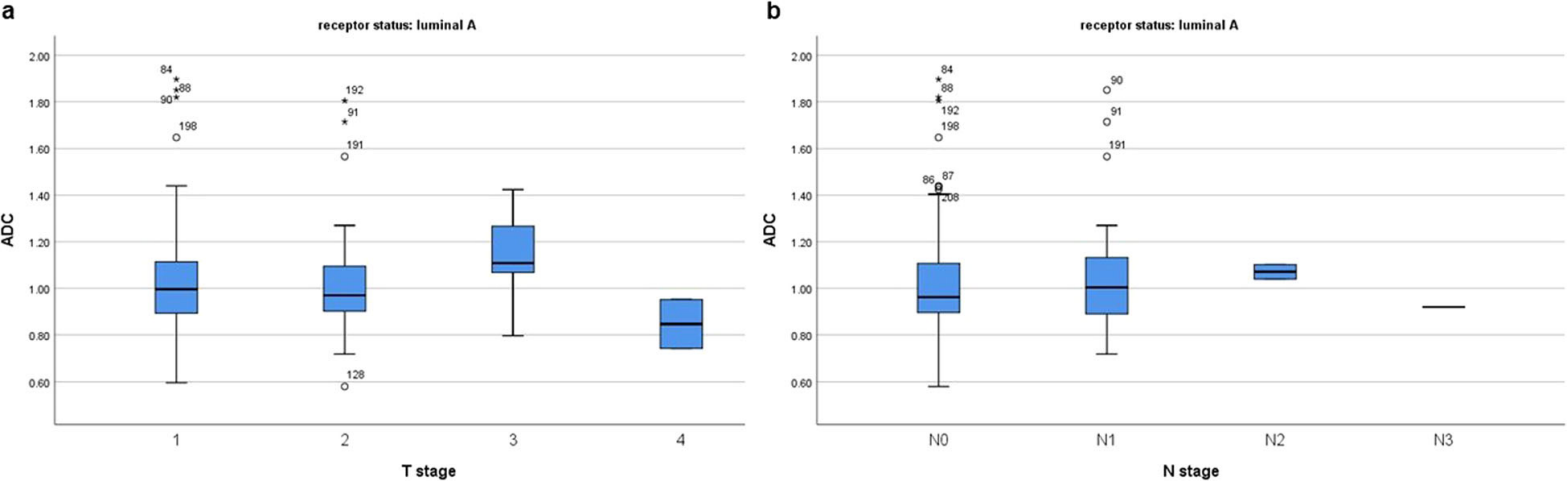
**a** Box plots of ADC values in different T stages of luminal A breast cancers. No statistical difference between the ADC values was identified (Kruskal-Wallis test *p* = 0.313). **b** Box plots of ADC values in different N stages of luminal A breast cancers (Kruskal-Wallis test *p* = 0.708)

**Fig. 5:**
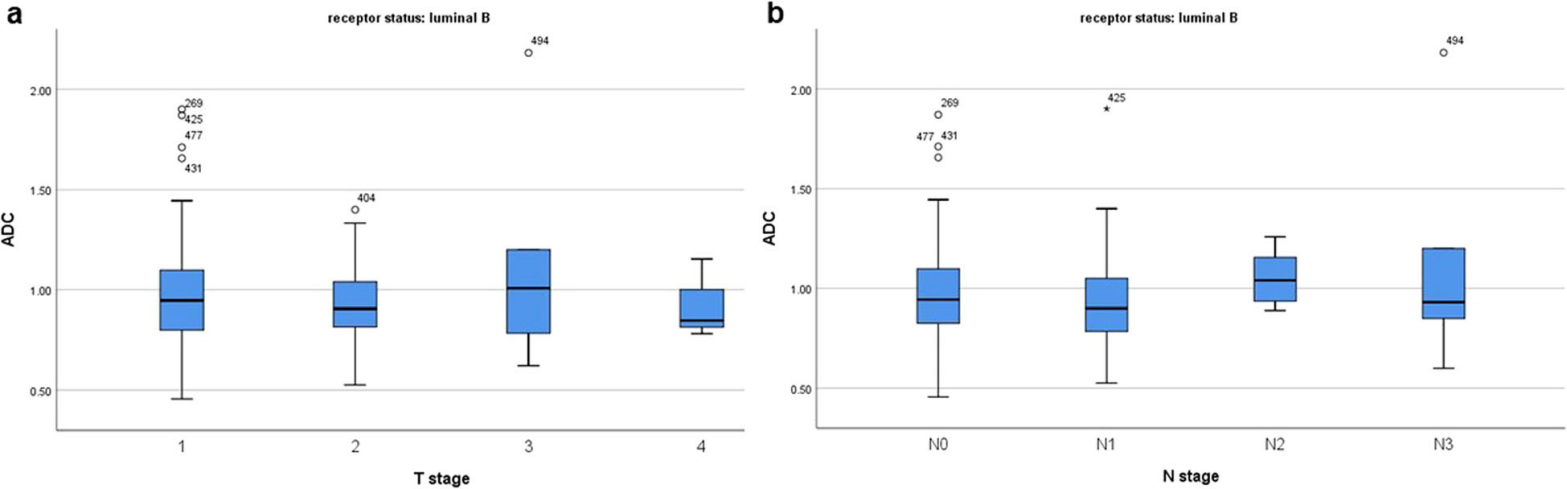
**a** Box plots of ADC values in different T stages of luminal B breast cancers (Kruskal-Wallis test *p* = 0.359). **b** Box plots of ADC values in different N stages of luminal B breast cancers (Kruskal-Wallis test *p* = 0.090)

**Fig. 6:**
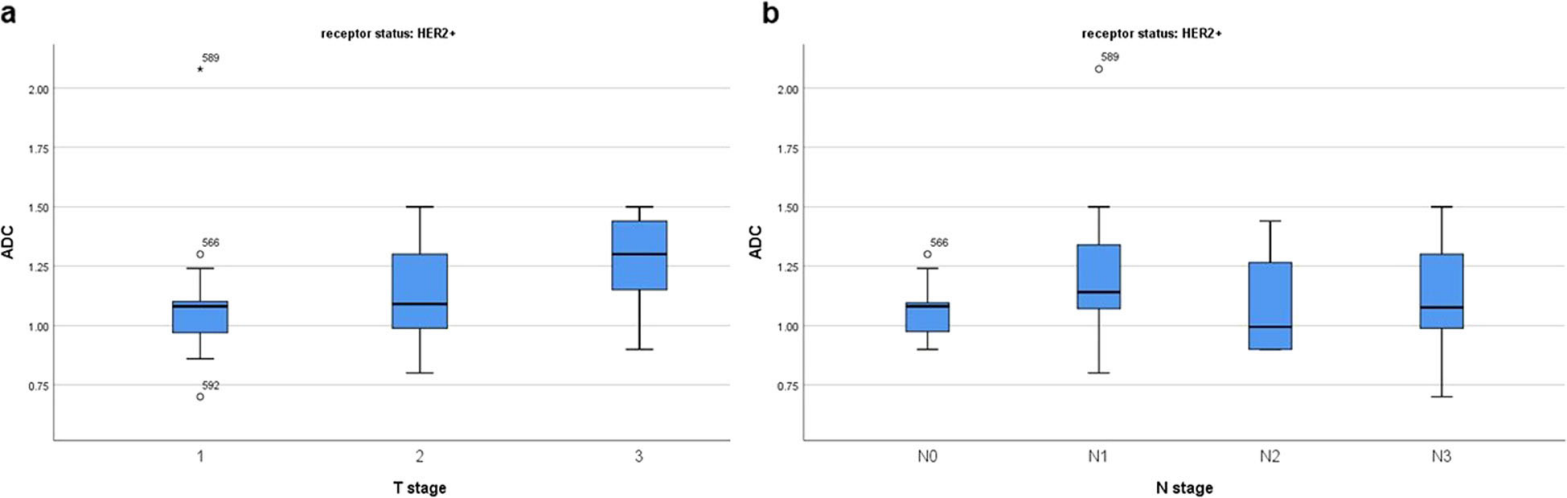
**a** Box plots of ADC values in different T stages of HER2+ breast cancers (Kruskal-Wallis test *p* = 0.233). **b** Box plots of ADC values in different N stages of HER2+ breast cancers (Kruskal-Wallis test *p* = 0.533)

**Fig. 7:**
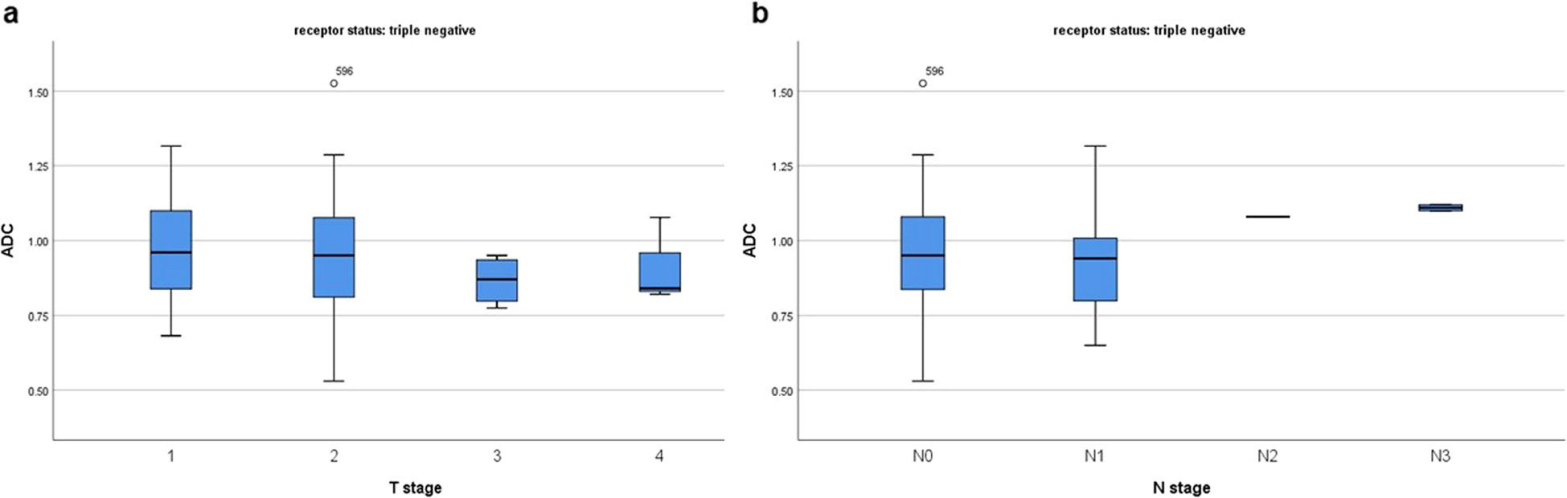
**a** Box plots of ADC values in different T stages of triple negative breast cancers (Kruskal-Wallis test *p* = 0.521). **b** Box plots of ADC values in different N stages of triple negative breast cancers (Kruskal-Wallis test *p* = 0.205)

Furthermore, also in the subgroups with different receptor status ADC could not predict T and/or N stage (Figs. 4, 5, 6, 7).

### ADC and expression of Ki 67

In overall sample, ADC correlated weakly with expression of Ki 67 (Table 3). Furthermore, also weak statistically significant correlation between ADC and Ki 67 was observed in luminal B carcinoma (*r* = − 0.130, *p* = 0.03). In luminal A, HER 2+ and triple negative tumors there were no significant correlations between ADC and Ki 67.

**Table 3:** Correlation between ADC values and expression of Ki 67 within molecular subtypes

Tumors	Correlation coefficients (*p* values)
Overall sample	***r*** **= −0.126 (*p*** **= 0.001)**
Luminal A	*r* = −0.063 (*p* = 0.402)
Luminal B	***r*** **= − 0.130 (*p*** **= 0.03)**
HER 2+	*r* = − 0.205 (*p* = 0.099)
Triple negative	*r* = 0.084 (*p* = 0.382)

Significant correlations are highlighted in bold

## Discussion

To the best our knowledge, this is the first multicenter study evaluating associations between ADC and prognostic pathologic factors in BC.

The possibility to reflect clinically relevant histopathological features may broaden the diagnostic horizon of MRI. If MRI, in particular ADC, can predict histopathology in BC, then ADC may be used as surrogate biomarker. Consequently, ADC may predict tumor biology and behavior, and, therefore, also tumor prognosis.

DWI measures diffusion of water molecules in tissues [16]. Numerous reports indicated that DWI can reflect several histopathological features of malignant and benign lesions [17–19]. It has been shown that ADC correlated inversely with cell count and proliferation index Ki 67 [17–19]. Furthermore, some authors suggested that ADC may be also associated with expression of epidermal growth factor receptor (EGFR) [20, 21], vascular endothelial growth factor (VEGF) [22], epidermal growth factor receptor 2 (HER2) [23], tumor suppressor protein p53 [20, 21], programmed cell death protein (PD L1) [24], nucleic content [25], and membrane permeability in several tumors [25]. Therefore, it might be possible that ADC may also depend on hormone receptor status in BC. As mentioned above, the results of the previously reported studies comparing ADC and hormone receptor status are contradictory and non-definitive. However, the present study based on a large multicenter sample showed that ADC cannot really discriminate tumors with different hormone receptor expression. Although, luminal B carcinomas had statistically significant lower ADC values in comparison to luminal A and HER 2+ BC, ADC values of different tumor subtypes overlapped significantly.

Another interesting aspect of the present study is the fact that ADC of primary tumors cannot predict lymph node status in BC. Previously, some reports indicated that ADC values of nodal metastasized BC were lower in comparison to non-metastasized tumors [26–28]. For example, Arponen et al. showed that lower ADC values correlated with presence of axillary metastases (*P* = 0.03) [26]. Our study did not confirm these results.

Similarly, we could not find any associations between T stage and ADC. This finding is well in agreement with previous reports, which also did not identify correlations between tumor size and ADC values in BC [27, 29].

Analysis of possible relationships between ADC and expression of Ki 67 is also a very important aspect of the present work. Besides hormone receptor, Ki 67 is one of the most clinically important biological markers in BC and can predict tumor prognosis, disease-free and overall survival [30, 31]. The reported data regarding associations between Ki 67 expression and ADC in BC are controversial. Recently, a large multicenter study could identify only weak correlation between ADC and Ki 67 (*p* = − 0.202, *P* < 0.001) [32]. This finding was in agreement with some meta analyses that also studied correlations between ADC and histopathological findings like proliferation potential and/or tumor cellularity [17, 19]. Therefore, it has been postulated that ADC cannot apply as surrogate biomarker for proliferation activity in BC [32]. We assumed, however, that different breast carcinomas may also show different associations between ADC and Ki 67. Similar phenomenon was previously identified in meningiomas [33]. Also in BC, it has been shown that different carcinoma subtypes namely invasive ductal carcinomas, invasive lobular carcinomas and ductal carcinoma in situ had different correlations between ADC and Ki 67 [32]. Furthermore, Mori et al. found that the mean ADC values correlated statistically significant (*r* = − 0.55, *P* < 0.0001) with Ki 67 in luminal BC [29]. However, according to Onishi et al., ADC did not correlate statistically significant (*r* = 0.035, *P* = 0.892) with Ki 67 in mucinous BC [34].

In fact, the present study showed that only in luminal B subtype ADC correlated statistically significant with Ki 67. However, the identified correlations were weak. In other subtypes, namely luminal A, HER 2+ and triple negative BC, no significant associations between the parameters were found.

There are some limitations to address. Firstly, this is a retrospective analysis. Secondly, different MR scanners with different technical parameters like field strength (Tesla), DWI sequences, and b values were used in different centers. Thirdly, the tumor cohort consisted predominantly of invasive ductal carcinomas. Furthermore, our cohorts had smaller number of HER2+ tumors in comparison to other subtypes.

All the mentioned factors may influence our results. However, our study is the largest to date and, therefore, provides evident data regarding associations between ADC and clinically relevant biological parameters in BC. Furthermore, our data reflects a real clinical situation.

## Conclusions

The present multicenter study showed that ADC is not able to discriminate molecular subtypes of BC, and cannot be used as a surrogate marker for disease stage or proliferation activity.

## Data Availability

The data that support the findings of this study are available from professor Surov but restrictions apply to the availability of these data, which were used under license for the current study, and so are not publicly available. Data are however available from the authors upon reasonable request and with permission of professor Surov.

## Abbreviations

ADC: apparent diffusion coefficient
BC: breast cancer
DWI: diffusion weighted imaging
MRI: magnetic resonance imaging
